# Non-antibody Approaches to Proprotein Convertase Subtilisin Kexin 9 Inhibition: siRNA, Antisense Oligonucleotides, Adnectins, Vaccination, and New Attempts at Small-Molecule Inhibitors Based on New Discoveries

**DOI:** 10.3389/fcvm.2018.00199

**Published:** 2019-01-29

**Authors:** Toshiyuki Nishikido, Kausik K. Ray

**Affiliations:** ^1^Imperial Centre for Cardiovascular Disease Prevention, Department of Primary Care and Public Health, School of Public Health, Imperial College London, London, United Kingdom; ^2^Department of Cardiovascular medicine, Saga University, Saga, Japan

**Keywords:** PCKS9 inhibition, LDL cholesterol, statin, monoclonal antibody, adverse effect

## Abstract

Low-density lipoprotein (LDL) is one of the principal risk factors for atherosclerosis. Circulating LDL particles can penetrate into the sub-endothelial space of arterial walls. These particles undergo oxidation and promote an inflammatory response, resulting in injury to the vascular endothelial wall. Persistent elevation of LDL-cholesterol (LDL-C) is linked to the progression of fatty streaks to lipid-rich plaque and thus atherosclerosis. LDL-C is a causal factor for atherosclerotic cardiovascular disease and lowering it is beneficial across a range of conditions associated with high risk of cardiovascular events. Therefore, all guidelines-recommended initiations of statin therapy for patients at high cardiovascular risk is irrespective of LDL-C. In addition, intensive LDL-C lowering therapy with statins has been demonstrated to result in a greater reduction of cardiovascular event risk in large clinical trials. However, many high-risk patients receiving statins fail to achieve the guideline-recommended reduction in LDL-C levels in routine clinical practice. Moreover, low levels of adherence and often high rates of discontinuation demand the need for further therapies. Ezetimibe has typically been used as a complement to statins when further LDL-C reduction is required. More recently, proprotein convertase subtilisin kexin 9 (PCSK9) has emerged as a novel therapeutic target for lowering LDL-C levels, with PCSK9 inhibitors offering greater reductions than feasible through the addition of ezetimibe. PCSK9 monoclonal antibodies have been shown to not only considerably lower LDL-C levels but also cardiovascular events. However, PCSK9 monoclonal antibodies require once- or twice-monthly subcutaneous injections. Further, their manufacturing process is expensive, increasing the cost of therapy. Therefore, several non-antibody treatments to inhibit PCSK9 function are being developed as alternative approaches to monoclonal antibodies. These include gene-silencing or editing technologies, such as antisense oligonucleotides, small interfering RNA, and the clustered regularly interspaced short palindromic repeats/Cas9 platform; small-molecule inhibitors; mimetic peptides; adnectins; and vaccination. In this review, we summarize the current knowledge base on the role of PCSK9 in lipid metabolism and an overview of non-antibody approaches for PCSK9 inhibition and their limitations. The subsequent development of alternative approaches to PCSK9 inhibition may give us more affordable and convenient therapeutic options for the management of high-risk patients.

## Introduction

Low-density lipoprotein particles account for 90% or more of plasma apolipoprotein B-containing atherogenic lipoprotein particles, hence LDL cholesterol (LDL-C) accounts for the majority of circulating cholesterol carried by atherogenic particles. LDL particles are able to penetrate the endothelium of the artery intima, leading to its oxidative modification into highly atherogenic particles that subsequently induces an inflammatory response. The uptake of oxidized LDL by macrophages converts them to foam cells initiating the process of atherosclerosis within the fatty streak. Genetic, observational and trial data demonstrate that LDL-C is a causal factor for atherosclerotic cardiovascular disease (CVD) and that lowering it reduces clinical events (1). Therefore, the clinical benefit of lowering LDL-C is widely acknowledged, and current guidelines recommend lipid-lowering strategies principally with statins for individuals at high cardiovascular risk (2–4). Reduction in LDL-C levels with 3-hydroxy-3methylglutaryl coenzyme A reductase inhibitors (statins) results in a dose-dependent reduction in LDL-C and of CVD risk, proportional to the absolute magnitude of the reduction in LDL-C levels. The Cholesterol Treatment Trialists' meta-analyses of data from 170,000 participants in 26 randomized trials involving intensive statin therapy revealed that 1 mmol/L (~40 mg/dl) reduction in LDL-C levels resulted in a 10% relative reduction in all-cause mortality (Relative risk (RR) 0.90, 95% confidence interval (CI) 0.87–0.93) and 22% relative reduction of major vascular events (non-fatal myocardial infarction, coronary death, coronary revascularization, or stroke) (RR 0.78, 95% CI 0.76–0.80) (5). Furthermore, achieving very low levels of LDL-C has beneficial effects on CVD risk, according to the meta-analysis of eight randomized controlled statin trials (6). In a *post-hoc* analysis of the JUPITER trial, the participants attaining LDL-C levels below 50 mg/dl with Rosuvastatin 20 mg experienced the fewest CVD events without an increase of the incidence of adverse events (7, 8). The IMProved Reduction of Outcomes: Vytorin Efficacy International Trial (IMPROVE-IT) demonstrated the incremental lowering of LDL-C levels by combining a non-statin drug with statin therapy (9). Among 18,144 patients who had experienced acute coronary syndromes, ezetimibe combined with statin therapy reduced the median time-adjusted average LDL-C level by 53.2 mg/dl after 1 year, and reduced the risk of a composite of cardiovascular death, major coronary event (non-fatal myocardial infarction, unstable angina, or coronary revascularization), or non-fatal stroke. These findings supported the notion that intensive LDL-C level reduction leads to improved outcome regardless of the lipid-modifying drug administered in combination with statins, particularly in high-risk patients (10). Observational data within the same study show that over a 7-year period those achieving the lowest LDL-C levels had the lowest risk and that such levels were safe. Life-long lowering of LDL-C levels resulting from genetic differences shows that the benefits of LDL-C lowering are cumulative as a genetically 13 mg/dl difference in LDL-C over 52 years offers the same reduction in risk as a 39 mg/dl over 5 years with statins. These data also suggest that that there may be benefits from early initiation of therapy (11, 12). Therefore, both the absolute magnitude of the reduction of LDL-C levels and the total duration of the period of low LDL-C levels should be considered when assessing the benefits of therapy.

Despite these findings, ~50% of patients treated with statins fail to achieve target the LDL-C levels recommended by the guidelines (13, 14). Moreover, 40% of the patients who receive high doses of statins do not achieve LDL-C levels below 70 mg/dl, even though patients with LDL-C levels below 50 mg/dl have a significantly lower risk of cardiovascular events than patients with LDL-C levels between 75 and 100 mg/dl. Hence, there is a large variation in the reduction in LDL-C levels in the general population. Risk factors do not exist in isolation, so risk factors such as diabetes mellitus, hypertension, abdominal obesity, smoking, etc., lead to higher absolute cardiovascular event risk when they occur together in a synergistic manner (15–17). This is highlighted further by the observation that individuals with established cardiovascular disease do not all have a 10-year risk of 20% but rather a wide variation in event rate. Part of this excess risk is because patients are not on optimal treatment and part of the need to reduce so called residual risk is to optimize control of all risk factors and achieve guideline-recommended treatment strategies (18). Even if guideline-based treatments were attained, there will be subsets of patients who by virtue of the risk factors they possess will require further LDL-C reduction or in whom LDL-C levels are high despite statins e.g., Familial hypercholesterolemia or who cannot tolerate therapies that are routinely available such as those with statin intolerance.

## PCSK9 as a Promising Therapeutic Target

**Figure 1:**
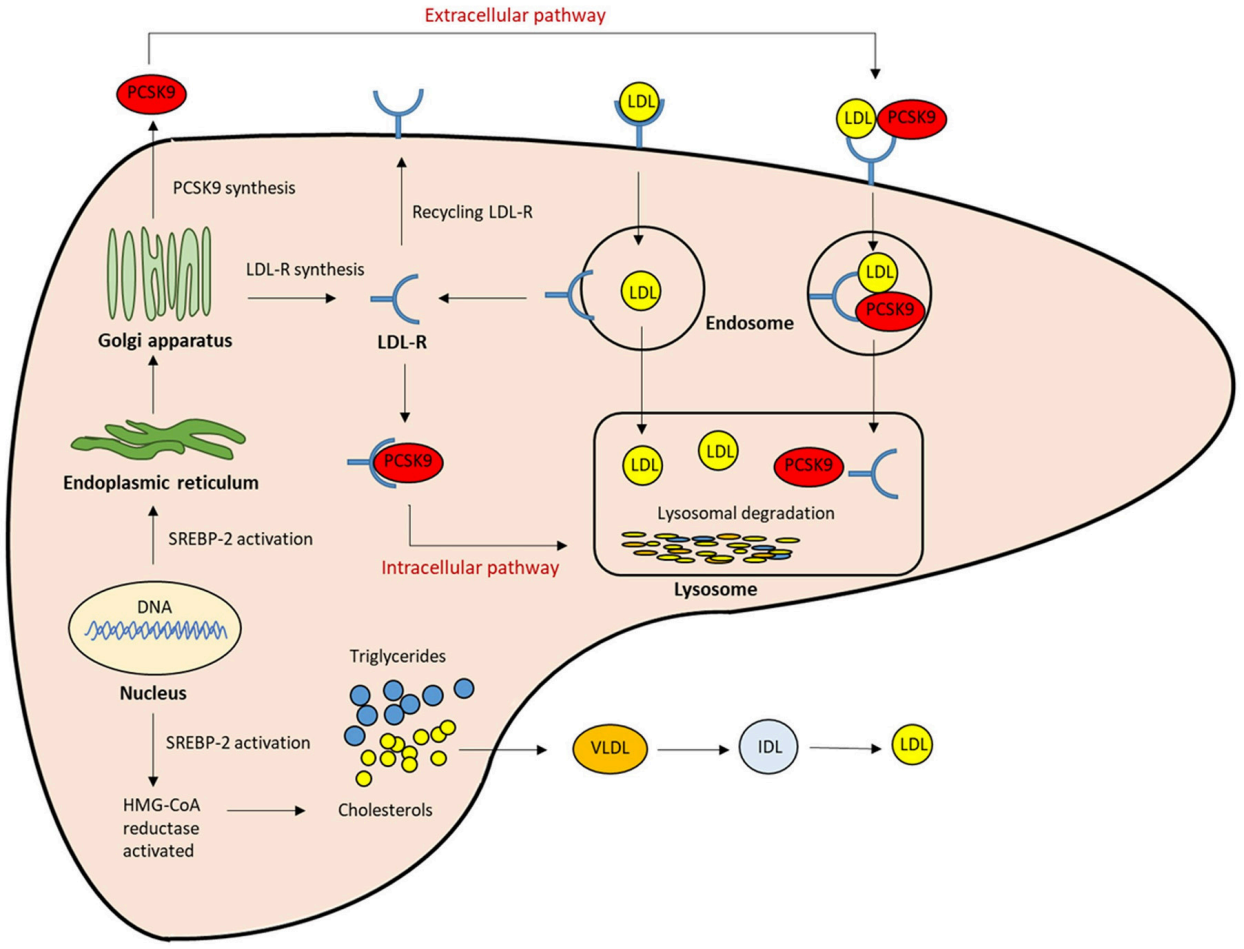
The role of PCSK9 in lipoprotein metabolism. The expression of LDLR and PCSK9 are regulated by SREBP-2. The PCSK9 secreted from the endoplasmic reticulum to the Golgi apparatus, and then from trans-Golgi network into the medium. It can be sorted directly to lysosomes as a complex with the LDLR (intracellular pathway), or secreted into the plasma and then internalized with the LDLR into clathrin-coated endosomes for lysosomal degradation (Extracellular pathway). Both mechanisms result in the reduction of LDLR, and then LDL clearance from the circulation is reduced.; LDL-R, LDL receptor; SREBP-2, Sterol regulatory element-binding protein-2; HMG-CoA, 3-hydroxy-3methylglutaryl coenzyme A.

The LDL receptor (LDLR) on the liver surface controls plasma LDL levels by binding to circulating LDL, and the LDL/LDLR complex is internalized by clathrin-mediated endocytosis. In endosomes, at acidic pH, LDL is released and degraded, while the LDLR is recycled to the cell surface (19). LDLR expression is predominantly regulated at the transcriptional level by a negative feedback mechanism in the intracellular cholesterol pool. Proprotein convertase subtilisin kexin 9 (PCSK9) plays an important role in the regulation of LDL-C homeostasis. PCSK9 binds to the LDLR, and thus promotes the endosomal and lysosomal degradation of the PCSK9-LDLR complex in a non-enzymatic fashion, and prevents LDLR recycling to the hepatocyte membrane. This leads to a reduction in LDL uptake and an increase in circulating LDL levels (20–22) (Figure 1). The mutations in the PCSK9 gene were identified in French families with autosomal dominant hypercholesterolemia in 2003, which were not due to either LDLR or apoB (23, 24). Rare gain-of-function PCSK9 mutations promoted degradation of LDLR, resulting in elevated LDL-C levels and increased CVD risk. On the other hand, more frequent loss-of function PCSK9 mutations were found to be associated with lower LDL-C levels and a reduced risk of CVD without adverse consequences (25–28). These findings suggest that the inhibition of PCSK9 may comprise a safe and effective strategy for addressing hypercholesterolemia. Hence, PCSK9 inhibition has emerged as a promising therapeutic target for the management of LDL-C.

### Structure and Function of PCSK9

PCSK9 is the ninth member of the subtilisin family of kexin-like proprotein convertases. It is expressed predominantly in the liver and, to a lesser extent, in the small intestine, kidney, pancreas, and the central nervous system (29, 30). The human 22-kb PCSK9 gene is located at chromosome 1p32, and contains 12 exons and 11 introns. It encodes a 692-amino acid serine protease (31). PCSK9 expression is mainly regulated transcriptionally, by the sterol regulatory element-binding protein (SREBP)-2, a membrane-bound transcription factor that controls cellular lipid homeostasis (32). Among the three identified isoforms of SREBP, SREBP-2 regulates genes involved in cholesterol homeostasis, such as LDLR, PCSK9, and HMGCoAR, whereas SREBP-1c regulates genes involved in fatty acid synthesis (33). It has been shown that *in vivo*, SREBP-2 increases the sterol-dependent transcription of PCSK9, predominantly via binding the sterol-regulatory element (SRE) (34). Moreover, PCSK9 expression is also regulated by hepatocyte nuclear factor (HNF)-1α, which is an essential positive regulator of PCSK9 transcription. The HNF-1α-binding site is located between SRE and Sp1 site, as a tissue-specific cis-regulatory sequence of the PCSK9 promoter (35, 36).

**Figure 2:**
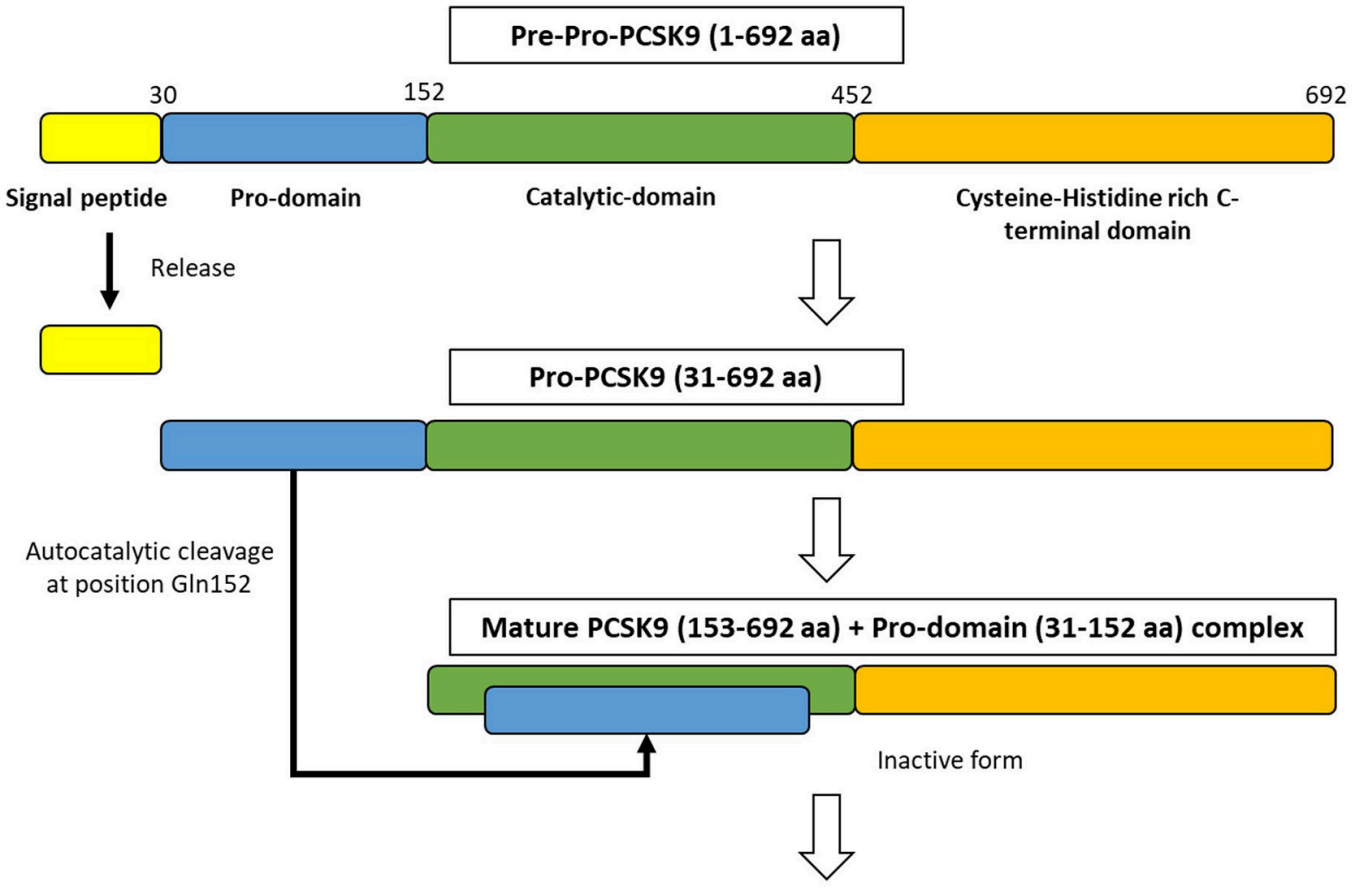
The synthesize of PCSK9 protein in the endoplasmic reticulum. PCSK9 comprises a single peptide (amino acid 1-30), a prodomain (amino acid 31-152), a catalytic domain (amino acid 153-452), and Cysteine-Histidine rich C-terminal domain (amino acid 453-692). In endoplasmic reticulum, proPCSK9 undergoes autocatalytic cleavage and the prodomain is separated from the mature PCSK9. The prodomain remains associated with the catalytic fragment, which inhibits the protease activity of the mature protein.; aa, amino acid.

PCSK9 is primarily synthesized as an inactive zymogen, proPCSK9 (74 kDa), composed of a single peptide with an N-terminal prodomain, a catalytic domain, and a cysteine- and histidine-rich C-terminal domain (21) (Figure 2). The C-terminal domain is composed of M1, M2, and M3 modules. M1 and M3 are associated with PCSK9 maturation and secretion, while M2 is required for intracellular LDLR degradation. In the endoplasmic reticulum (ER), the signal peptide of proPCSK9 is cleaved and the peptide undergoes co-translational autocatalytic cleavage at position Gln152 into mature PCSK9 (63 kDa). The cleaved prodomain (14 kDa) remains non-covalently bound to the catalytic domain. It subsequently acts as a chaperone to assist the proper folding of mature PCSK9, which is required for PCSK9 to exit from the ER. In addition, it blocks the catalytic site of mature PCSK9 enzymatically through the secretory pathway (37, 38). Thus, PCSK9 is secreted from the ER to the Golgi apparatus as an inactive mature PCSK9/prodomain complex, which prevents the binding of any other proteins or peptides to the catalytic sites and inhibits the protease activity of PCSK9 (32, 39–41). However, this complex can bind to specific target proteins and escort them to intracellular degradation compartments (42). The C-terminal domain of PCSK9 acts as a chaperone for PCSK9 mediated LDLR degradation (43–46). The LDLR ectodomain consists of the N-terminal ligand-binding domain and the epidermal growth factor (EGF) precursor homology domain (EGF-A, EGF-B, -propeller, and EGF-C domains). Thus, the PCSK9 catalytic domain directly binds to the first EGF-A domain of the LDLR on the cell surface (47, 48). On the surface of hepatocyte plasma membrane, the catalytic domain of secreted PCSK9 is internalized and the LDLR/PCSK9 complex enters the endosomal pathway. In the acidic compartment of the endosome, the affinity of PCSK9 toward LDLR is enhanced, while LDL is released from the LDLR. Thus, PCSK9 disrupts the recycling of LDLR to the cell membrane and acts to increase the plasma LDL levels. Furthermore, PCSK9 targets the LDLR for degradation not only via the extracellular pathway but also the intracellular pathway. PCSK9 also enhances intracellular LDLR degradation without recycling to the cell surface by direct intracellular trafficking from the trans-Golgi network to the late endosomes/lysosomes. This pathway requires clathrin light chains, and involves a different sorting mechanism than that of the extracellular degradation pathway (32, 49) (Figure 1).

### PCSK9 Monoclonal Antibodies (MAbs)

**Figure 3:**
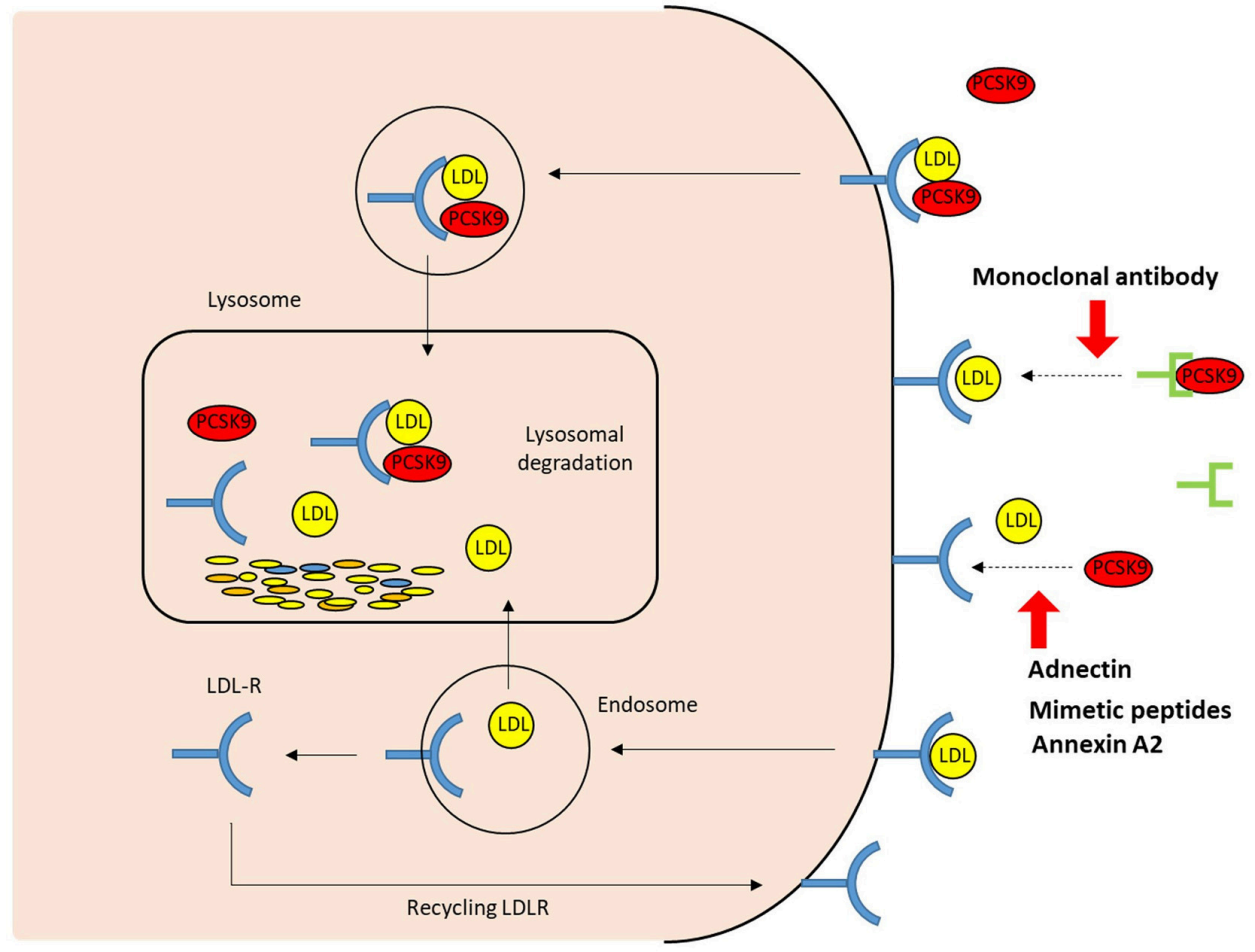
The extracellular mechanisms of PCSK9 inhibition. The strategies that target the binding between PCSK9 and LDLR extracellularly include PCSK9 monoclonal antibodies, adnectin (BMS-962476), mimetic peptides, and small molecules. By blocking PCSK9, these PCSK9 inhibitors increase LDLR recycling and LDL uptake; LDLR, LDL receptor.

The first approach to inhibit PCSK9 involved inhibiting the binding of PCSK9 to the LDLR (Figure 3). Two fully human anti-PCSK9 monoclonal antibodies (MAbs), alirocumab (SAR236553/REGN727 from Regeneron Pharmaceuticals/Sanofi) and evolocumab (AMG145 by Amgen), are currently approved for the treatment of hypercholesterolemia by the US Food and Drug Administration and European Medicines Agency. Anti-PCSK9 MAbs bind the catalytic domain of PCSK9, blocking extracellular interaction with the EGF-A domain of the LDLR by neutralizing PCSK9 (50). Subcutaneous injection of anti-PCSK9 MAbs provides a rapid and persistent reduction of LDL-C levels. Numerous trials and subsequent meta-analyses demonstrate that anti-PCSK9 MAbs reduce LDL-C levels by ~50–60% in patients with a diet-based therapy, alone or with various doses of stains without serious adverse events. Moreover, treatments involving PCSK9 MAbs have been shown to improve cardiovascular outcomes (51–55).

Currently, two large-scale cardiovascular outcome trials have been conducted to evaluate the efficacy of the two PCSK9 MAbs mentioned above. The Further Cardiovascular Outcomes Research with PCSK9 inhibition in Subjects with Elevated Risk (FOURIER) study involving 27,564 high-risk patients with stable atherosclerotic cardiovascular disease is a large cardiovascular outcome clinical trial of the PCSK9 inhibitor evolocumab (56). The combination of evolocumab with statin therapy resulted in a substantial 59% reduction in LDL-C levels from a mean baseline value of 92–30 mg/dl, and a 15% relative reduction of cardiovascular events (cardiovascular death, myocardial infarction, stroke, hospitalization for unstable angina, or coronary revascularization) after a median follow-up of 2.2 years.

The ODYSSEY OUTCOME trial enrolled 18,924 patients who had experienced a recent acute coronary syndrome and with elevated LDL-C levels despite high-intensity statin therapy (57). A reduction in LDL-C levels by 61% compared to placebo after one year was observed even though ~90% of patients were receiving high intensity statin therapy. Alirocumab reduced major adverse cardiovascular events (coronary heart disease death, non-fatal myocardial infarction, fatal or non-fatal ischemic stroke, and unstable angina requiring hospitalization) by 15% (hazard ratio: 0.85, 95% CI: 0.78–0.93) and all-cause death by 15% (hazard ratio: 0.85, 95% CI: 0.73–0.98). Taken together these two outcome trials demonstrate the effectiveness of PCSK9 inhibition in high risk populations with LDL-C > 70 mg/dl despite maximally tolerated/high intensity statin therapy.

There are issues which limit the widespread use of anti-PCSK9 MAbs. Anti-PCSK9 MAbs need to be administered by subcutaneous injection once or twice a month. The dosing frequency is low compared with other injectable treatments, such as insulin therapy. At the current dosing frequency, a high level of adherence over at least one year was reported for the self-injected anti-PCSK9 MAbs in a pooled analysis of six clinical trials of alirocumab (58). However, the use of injectable PCSK9 agent for essentially asymptomatic patients has several considerations; firstly maintaining the recommended schedule of injections over the long-term, and adequate refrigeration are required. Hence, it is unclear whether the adherence to these agents is any higher than that of daily administration of oral drugs in the real-world setting. In addition, PCSK9 inhibitors are not likely to be cost-effective for all patients given their current high price. It is estimated that the cost of PCSK9 inhibitors was ~$14,000 per person per year in the US and $5,000–7,000 per person per year in Europe (59–61). Specific high-risk sub-groups of patients with established vascular disease and in particular such patients with additionally a high baseline LDL-C would be expected to derive the greatest benefit and consequently these treatments are more cost-effective in such cases. Currently, the plan to cut the price of PCSK9 MAbs in the US have been announced by the pharmaceutical companies. Improving the cost effectiveness may provide an effective treatment option for a broader patient population. Whilst PCSK9 is a validated target for treatment there are several limitations to MAbs and recently, several approaches to inhibiting PCSK9 are under development as described below.

## Non-antibody Approaches to PCSK9 Inhibition

**Figure 4:**
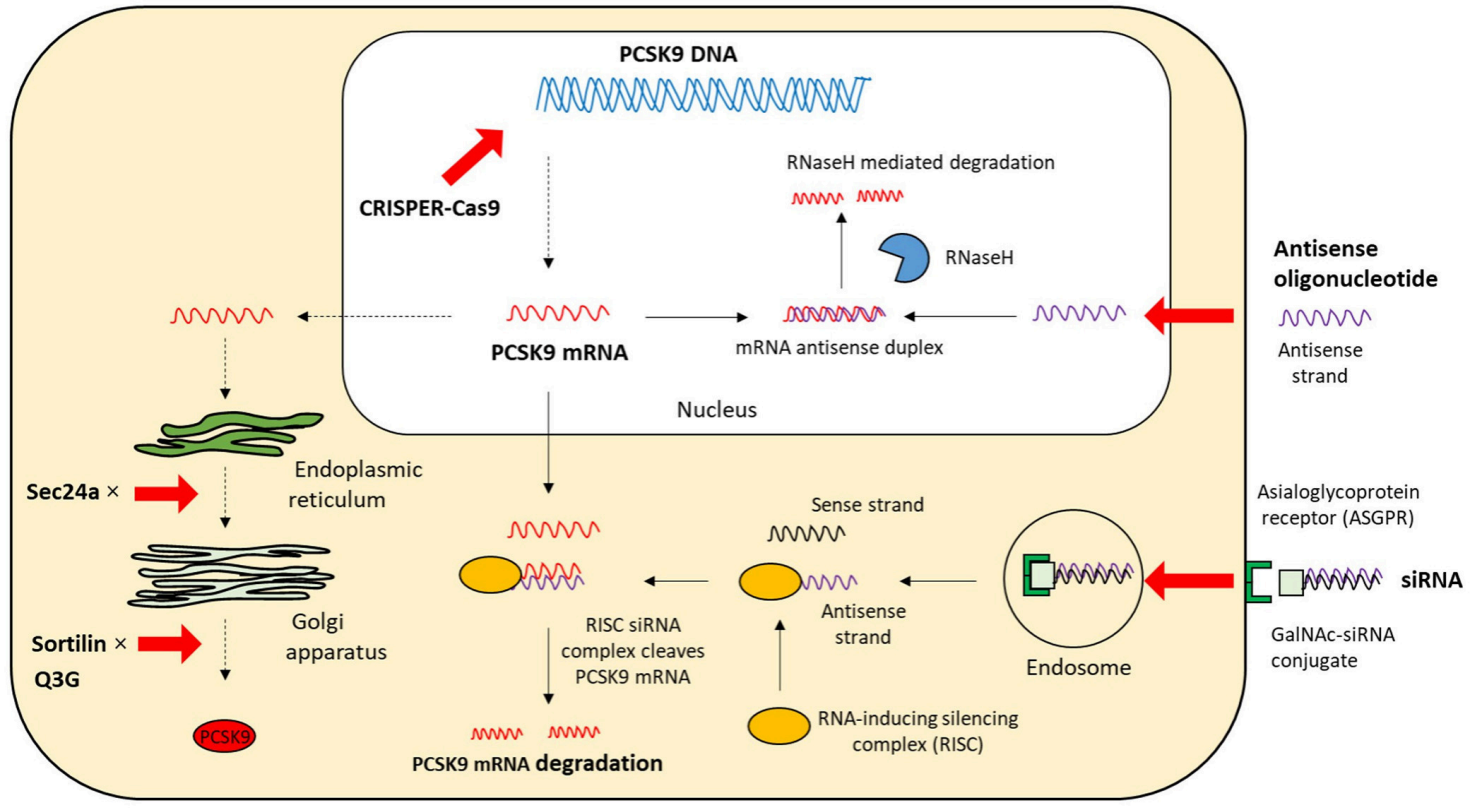
The intracellular mechanisms of PCSK9 inhibition. The strategies that target the expression and secretion of PCSK9 intracellularly include antisense oligonucleotide, siRNA, CRISPR/Cas9 gene editing, and small molecules; mRNA, messenger RNA; siRNA, small interfering RNA; CPRISPER-Cas9, Clustered regularly interspaced short palindromic repeat (CRISPR)/CRISPR-associated protein 9; GalNAc, N-acetylgalactosamine; Q3G, quercetin-3-O-beta-glucoside; RNaseH, Ribonuclease H.

Based on the biology of PCSK9 inhibition of PCSK9 expression, secretion from cells and interaction between PCSK9 and LDLR are considered to be effective and alternative approaches to MAbs (Figures 3, 4). The current therapeutic options involve gene editing or silencing technologies, small-molecule inhibitors, mimetic peptides, and vaccination (Table 1).

**Table 1:** The comparison among different approaches to PCSK9 inhibition.

	**MAbs**	**siRNA**	**ASO**	**Small molecules**	**Mimetic peptides**	**Adnectin**	**Vaccination**
Structure	Monoclonal antibody	Double-stranded RNA	Single-stranded RNA	Protein	Peptide	Protein	Peptide
Administration	Once or Twice monthly, Subcutaneous injection	Twice yearly, Subcutaneous injection	Monthly, Subcutaneous injection	Daily, Oral administration	Injection administration	Subcutaneous or intravenous injection	Once yearly, Subcutaneous injection
Mechanism to action	Blocking the extracellular interaction of PCSK9 with LDLR	PCSK9 synthesis inhibition through RNA interference	PCSK9 synthesis inhibition through RNA interference	Blocking the intracellular or extracellular interaction between PCSK9 and enzyme or receptor	Blocking the extracellular inter action between PCSK9 and LDLR	Blocking the extracellular interaction of PCSK9 with LDLR	Eliciting production of autoantibodies against PCSK9
Efficacy	LDL-C 60%, PCSK9 60% reduction	LDL-C 50%, PCSK9 70% reduction	LDL-C 25–50%, PCSK9 50–85% reduction (Clinical + preclinical results)	LDL-C 50–60% reduction	LDLR 50% reduction (Preclinical results)	LDL-C 50%, PCSK9 > 90% reduction	LDL-C 50%, PCSK9 60% reduction (Only preclinical results)
Advantage	High specificity, No serious adverse reaction	High specificity, Infrequent dosing, Long-term effect, No serious adverse reaction	High specificity	Oral administration, Easy production, Low cost	High specificity, Easy production	High specificity, Easy production, Low cost	Long-term effect, Infrequent dosing, Easy production, Low cost
Disadvantage	Frequent subcutaneous dosing, Short shelf life, High cost	Subcutaneous dosing	Serious renal adverse event	Low selectivity, Non-tissue specific effect	Intravenous dosing	Frequent subcutaneous or intravenous dosing, Short half-life	Subcutaneous dosing
Current phase	Approved	Phase 3 on going	Phase 1 (terminated)	Phase 1	Preclinical	Phase 1	Phase 1 on gong
References	(51, 52)	(67)	(74, 75)	(90, 91)	(107–112)	(119, 120)	(122–124)

*MAbs, monoclonal antibody; siRNA, small interfering RNA; ASO, antisense oligonucleotide; LDLR, LDL receptor*.

### Interfering With PCSK9 Expression

#### Small Interfering RNA (siRNA)

RNA interference is an endogenous post-transcriptional mechanism for regulating gene expression in almost any type of cell. It is specifically mediated by small interfering RNA (siRNA). siRNAs are double-stranded RNA molecules (~20–25 bp long), consisting of two short-stranded RNA molecules, a passenger sense strand, and a guide (anti-sense) strand that is complementary to the target mRNA. The siRNA duplex is separated by an enzyme called DICER and the guide anti-sense strand is loaded onto the RNA-induced silencing complex, which leads to sequence-specific gene silencing by catalytic cleavage and degradation of the targeted mRNA (62, 63). The single antisense strand/ RISC complex is highly stable hence multiple mRNA transcripts can be catalytically degraded.

siRNA molecules can be synthesized relatively easily and must be modified to prevent degradation by endonucleases. ALN-PCS, a PCSK9-specific siRNA formulated in a lipid nanoparticle, was developed as a PCSK9 inhibitor. This new approach resulted in a rapid and durable reduction of PCSK9 mRNA and LDL-C levels. Healthy volunteers received ALN-PCS by intravenous infusion for 60 min. The highest dose of ALN-PCS reduced PCSK9 mRNA and protein levels by 70% and LDL-C levels by 40% after 3 days. The formulation of ALN-PCS was subsequently improved to limit the dose and volume of drug injected (64). Inclisiran (ALN-PCSsc: ALN-60,212 by Alnylam/The Medicines Company) is a fully chemically modified stabilized PCSK9-specific siRNA. Inclisiran is composed of one 2′-deoxy, eleven 2′-fluoro-, and thirty-two 2′-O- methyl-modified nucleotides, and a triantennary N-acetylgalactosamine (GalNAc) that is conjugated to the 3′-end of the passenger strand. The asialoglycoprotein receptor expressed highly on the hepatocyte surface specifically recognizes the GalNAc (65), which enables a rapid uptake of inclisiran with high specificity, only by the liver, after a subcutaneous low-volume injection. The plasma levels of inclisiran thus fall to undetectable levels within 24 h (66). This mechanism effectively prevents any off-target effects because PCSK9 is also present in extrahepatic tissues.

In phase II of the placebo-controlled, double-blind, randomized trial (ORION-1 trial), the efficacy, safety, and tolerability of inclisiran were evaluated in patients who had a history of atherogenic CVD or high atherosclerotic CVD-risk with elevated LDL-C levels despite having received the maximum tolerated doses of lipid-lowering therapy (67, 68). Patients who received two doses of inclisiran (300 mg per dose) on days 1 and 90 exhibited mean percent reductions of the PCSK9 and LDL-C levels of 69.1 and 52.6%, respectively, on day 180. Furthermore, no significant serious adverse events induced by inclisiran were apparent, and thus it was deemed to be relatively safe and well-tolerated during the study. These observations suggest that the administration of inclisiran by the healthcare professionals once or twice a year might result in potent and long-lasting lowering of LDL-C levels. This may improve treatment adherence and individual variability in response to the treatment. However, the mechanism of the long-lasting effect remains unknown and, hence, the effect of inclisiran should be investigated in a pharmacokinetic study. Further, safety monitoring over a long period of time in further clinical studies is also needed. Currently, there are some ongoing phase III trials (ORION 4, 5, 9, 10, and 11) for a large number of patients with the risk of atherosclerotic CVD or familial hypercholesterolemia. ORION-4 trial will evaluate the effect of inclisiran on cardiovascular outcomes in approximately 15,000 participants with pre-existing atherosclerotic CVD.

#### Antisense Oligonucleotides (ASOs)

ASOs, which consist of short, single-stranded nucleotides (12–25 bp long), interfere with gene expression by binding to the target mRNA directly, via Watson-Crick base paring, within the nucleus or cytoplasm. The majority of currently developed ASO drugs rely on an RNase H-dependent cleavage to degrade mRNA in a DNA-RNA hybrid (69). The activity of RNase H-dependent ASO is different between nucleus and cytoplasm due to some factors such as specificity and concentration of RNase H protein (70, 71). ASOs against PCSK9 mRNA inhibit protein synthesis to specifically reduce intracellular and extracellular PCSK9 levels.

Second-generation ASOs targeting PCSK9 mRNA (ISIS 394814/BMS 844421 by Bristol Myers Squibb/ISIS Pharmaceutical), with an affinity-enhancing modification of a nucleic acid-building block called, 2′-O-methoxyethyl RNA, were initially explored in preclinical studies. It was demonstrated that the administration of high doses of 2-O-methoxyethyl RNA ASOs lowered PCSK9 mRNA levels by 92% and increased LDLR protein levels 2-fold, which resulted in a reduction of LDL-C levels by 38% *in vivo* (72). However, the development of these ASOs was terminated because of insufficient binding affinity. Subsequently, shorter ASOs (SPC4061/SPC5001 by Santaris Pharma A/S) relying on locked nucleic acid technology is being developed instead of second-generation ASOs. These ASOs targeting PCSK9 have a stable conformation, and higher binding affinity and specificity for PCSK9 mRNA, inducing rapid, sustained, and potent effects compared with longer oligonucleotide. Early studies suggest that PCSK9 mRNA levels are reduced by 60% within 24 h post-injection, and the effect was sustained for over 16 days (73). In non-human primates, the hepatic PCSK9 mRNA levels and plasma LDL-C levels decreased by 85 and 50%, respectively (74). Meanwhile, subcutaneous administration of SPC5001 lowered the PCSK9 mRNA and LDL-C levels in a dose-dependent manner, by ~50 and 25%, respectively, in healthy human volunteers. The drug also decreased apolipoprotein B and increased apolipoprotein A1 levels (75). Nevertheless, transient renal tubular toxicity (one subject experienced an onset of acute tubular necrosis) and injection site reactions were also observed, and further clinical development of SPC4061/SPC5001 was terminated (75, 76). The exact cause of acute kidney also injury remains uncertain. Hence, understanding the molecular mechanism of renal toxicity and a strategy for sensitive detection of renal injury are important in the future development of this oligonucleotide drug. As another example, an anti-PCSK9 antisense oligonucleotide modified by a bridged nucleic acid is being developed; in early studies it exhibited high potency and low toxicity. In mice fed an atherogenic diet and administered this compound twice weekly for 6 weeks, a dose-dependent reduction of PCSK9 mRNA and LDL-C levels was observed (77).

The technology of antisense oligonucleotide synthesis is progressing by developing various structural modifications of the phosphate backbone, sugar moieties, and targeting ligand (78). These allow low frequency of administration and low individual variations compared with other small-molecule drugs. However, these modifications might cause potential off-target effects or adverse effects (79). It is important that ASOs accurately match the target mRNA, and are specifically delivered to the hepatocytes to reduce off target gene toxicity or effects in other tissues which may be undesirable.

#### Clustered Regularly Interspaced Short Palindromic Repeat (CRISPR)/CRISPR-Associated Protein 9 (Cas9)

CRISPR/(Cas9) (by Academic project/AstraZeneca) is a useful genome-editing tool that allows a quick destruction or editing of a target gene. This system is more accurate, faster, and cheaper than other existing genome-editing tools. It consists of Cas9 endonuclease and single guide RNA (sgRNA), which is ~100 bp long. A 20-nt sequence at the 5′-end of sgRNA recognizes and hybridizes with a complementary sequence of target DNA site located directly next to a protospacer-adjacent motif sequence (80). The Cas9-sgRNA–mediated DNA cleavage generates a double-strand break that triggers DNA repair machinery by non-homologous end-joining or homology-directed repair (81). Non-homologous end-joining is error-prone and induces disruptive insertions and/or deletions at the target site, while homology-directed repair can use an exogenous DNA repair template to perform precise knock-in of a desired alteration of the genomic DNA (82, 83). The CRISPR/Cas9 system has been shown to effectively disrupt the PCSK9 gene in mouse liver, resulting in significantly reduced plasma PCSK9 protein levels and increased hepatic LDLR levels. The plasma cholesterol levels were reduced by 35–40%, which was consistent with the 35–52% reduction observed in PCSK9 knockout mice compared with wild-type mice (84–86). Non-sense mutations resulting in loss-of-function of PCSK9 are associated with significant reduction of both LDL-C levels and coronary heart disease risk, with no adverse clinical consequences (28). This implies that PCSK9 genome editing technique might be a novel strategy that may be used to permanently alter the human genome for the prevention of coronary heart disease in patients with hypercholesterolemia.

However, the genome-editing technology entails ethical, legal, and safety issues that need to be resolved before it can be used as a general widely accepted therapeutic approach in human gene (87). The major limitation of CRISPR-Cas9 editing is more frequent off-target mutation than the intended mutation. The targeting specificity of Cas9 is regulated by a connection between the guide sequence of sgRNA and the protospacer-adjacent motif next to the target sequence in the genome. However, off-target cleavage is observed on a DNA sequence that harbors several mismatches with the distal protospacer-adjacent motif in the sgRNA-guiding sequence (88). It is important to detect potential off-target sites and optimize the CRISPR/Cas9 system to reduce the off-target activity without sacrificing the efficiency of on-target cleavage and minimize off-target mutations. Currently, CRISPR-Cas9 technology is widely adapted in various fields as a promising genome-editing tool that is easy to use and characterized by good cost-effectiveness. The long-term benefit and safety in human subjects need to be assessed by designing sgRNA with higher specify than currently attainable.

#### Small Molecules

Cholesteryl ester transfer protein (CETP) under normal physiological conditions transfers equimolar amount of triglycerides to high-density lipoprotein, while it promotes the transport of cholesteryl ester from high-density lipoprotein to apolipoprotein B-containing lipoproteins, such as very low-density lipoprotein, intermediate-density lipoprotein, and LDL (89). Hence, CETP inhibitor pharmacologically increases high-density lipoprotein-cholesterol levels and decreases LDL-C levels. A new CETP inhibitor, called K-312 (by Kowa), reduces PCSK9 expression in hepatocytes by decreasing the active forms of SREBP-1 and SREBP-2 that regulate PCSK9 promoter activity (90). *In vivo*, cholesterol-fed white rabbits were administered K-312 for 2 weeks which resulted in a 63% reduction of PCSK9 mRNA levels in the liver, reduced LDL-C levels (vehicle vs. K-312: 272.5 ± 42.2 mg/dl vs. 193.0 ± 36.5 mg/dl), and increased high-density lipoprotein-cholesterol levels (vehicle vs. K-312: 29.9 ± 3.7 mg/dl vs. 93.3 ± 14.7 mg/dl). Even though the plasma CETP activity was suppressed by K-312, the suppression of PCSK9 mRNA expression by K-312 was observed after siRNA silencing of CETP *in vitro*. Therefore, K-312 can decrease not only LDL-C levels but also PCSK9 expression via a mechanism independent of CETP inhibition. In addition, K-312 treatment has been shown to attenuate the progression of atherosclerosis. Hence, this compound may serve as a new effective therapy for hypercholesterolemia and possibly prevention of CVD.

PF-06446846 (by Pfizer) is an orally active small molecule, which directly and selectively inhibits translation of PCSK9 by stalling the 80 S ribosome in the proximity of codon 34. Its activity is dependent on the amino acid sequence of the nascent chain within the ribosome exit tunnel. Orally-administered PF-06446846 reduces plasma PCSK9 and LDL-C levels without liver toxicity symptoms *in vivo* (91). This promising approach had the potential of becoming a potent therapeutic option inhibiting PCSK9 synthesis by oral dosing; however, its development was discontinued for competitive reasons (92).

Berberine (BBR) is a natural cholesterol-lowering compound. It is an isoquinoline plant alkaloid (2,3-methylenedioxy-9,10-dimethoxyprotoberberine chloride) isolated from Huanglian (Coptis chinensis), and exerts different pharmacological and therapeutic effects on carbohydrate and lipid metabolism, diabetes mellitus, the immune system, endothelial function, and cardiovascular system, etc. (93). Previous studies indicated that BBR up-regulates the expression of LDLR-coding gene independent of intracellular cholesterol levels and down-regulates PCSK9 transcription. It has been demonstrated that oral administration of BBR reduced plasma levels of total cholesterol by 29%, triglycerides by 35%, and LDL-C by 25% in 32 patients with hypercholesterolemia (94). PCSK9 synthesis is controlled at transcriptional level by SREBPs and HNF-1α, which work cooperatively with SREBP-2 to transactivate the PCSK9 promoter (35). BBR simultaneously decreases HNF-1α and SREBP-2 levels, leading to a strong suppression of PCSK9 transactivation. Further, *in vitro*, BBR reduces PCSK9 mRNA levels in a time- and dose-dependent manner (95). This mechanism may negatively affect LDL-C metabolism because SREBPs are also important for increasing LDLR transcription; however, BBR was found to elevate LDLR expression independent of SREBPs via a post-transcriptional mechanism that stabilizes the LDLR mRNA *in vivo* (94). Nevertheless, further studies into the precise mechanism of BBR inhibition of PCSK9 are needed, before BBR may progress to the next stage of drug development as a new therapeutic molecule for lowering LDL-C levels.

Oleanolic acid is a pentacyclic triterpenoid that is common in nature, and widely distributed in plants and medicinal herbs as a free acid or as a saponin aglycone (96). It exerts various beneficial effects, such as anti-cancer, hepatoprotective, hypolipidemic (97), anti-oxidative, anti-inflammatory (98, 99), and endothelial-protective effects (100). Previous reports demonstrate that oleanolic acid treatment reduces total serum cholesterol, LDL-C, triglyceride, and free fatty acid levels in mice. Moreover, oleanolic acid reduced PCSK9 protein and mRNA levels in a time- and dose-dependent manner *in vitro* (39). However, the mechanism of PCSK9 inhibition and the utility as a PCSK9 inhibitor remain unknown.

All small-molecule inhibitors have the advantage of oral administration and lower production cost than MAbs. However, the achievement of oral delivery will require the maintenance of stability and potency to avoid proteolytic degradation in the gastrointestinal tract. In addition, broad blood distribution due to oral administration may increase the possibility of harmful side effects because of off-target interactions, since it is less specific than MAbs.

### Blocking the Function of PCSK9

#### Small Molecules

Oral administration of small-molecule inhibitors specific to PCSK9 appears to constitute a promising therapeutic strategy to disrupt the interaction of PCSK9 and LDLR. The small-molecule inhibitors can be produced easily and inexpensively. However, this approach remains challenging because it is difficult to design small molecules that specifically bind to the site of interaction between the catalytic subunit of PCSK9 and the EGF-A domain of LDLR because of the fairly large size of the flat interface (47, 101). In addition, small molecules have generally poor stability, selectivity, and potency. Small molecule-inhibitors of PCSK9 might be less specific than PCSK9 MAbs which increase the potential of adverse drug reactions and allosteric effects on different intracellular functions of PCSK9.

In spite of the above, DS-9001a (by Daiichi Sankyo/Pieris Pharmaceuticals), a small biologic molecule (~22 kDa) which requires subcutaneous administration, was recently created. DS-9001a is composed of an albumin-binding domain fused with an artificial lipocalin mutein (ABD-fused anticalin protein). The lipocalin architecture provides a high structural plasticity of the binding site, and enables potent and specific recognition of the target protein (102). DS-9001a strongly inhibits the binding of PCK9 to LDLR and attenuates LDLR degradation. A single intravenous injection of DS-9001a resulted in a reduction of LDL-C levels by ~62% and this effect was sustained for up to 21 days in the cynomolgus monkey (103). It can be manufactured using a bacterial expression system, which is associated with a low production cost. In addition, the small size and high solubility of DS-9001a enable the administration of increased drug concentrations at lower volumes than those of MAbs (104). A single ascending-dose study of DS9001a performed its safety and tolerability in healthy volunteers. Further, pharmacokinetic/pharmacodynamic analysis elucidated a dose-dependent reduction of free PCSK9 and LDL-C levels. Effective half-life of DS-9001a was ~12.5 days (105). Therefore, DS-9001a may constitute a potent therapeutic option, as an alternative to anti-PCSK9 MAb therapy.

#### Mimetic Peptides

Mimetic peptides are small amino acid stretches designed to biologically mimic the peptic structure of a target protein. Several peptides that mimic the EGF-A or EGF-AB binding domains of LDLR have been developed as competitive PCSK9 inhibitors, which bind to the catalytic domain of PCSK9, and limit the interaction between PCSK9 and LDLR. Peptides have emerged as therapeutic alternatives that bridge the gap between small molecules and large antibodies (106). A synthetic LDLR EGF-A domain peptide has been found to inhibit PCSK9-mediated degradation of LDLR and maintain LDL uptake in a dose-dependent manner (44, 107). Furthermore, recently a truncated 26 amino acid EGF-A analog, which is a smaller peptide-based inhibitor of PCSK9/LDLR binding, has been synthesized (108). This exhibits an enhanced binding affinity to PCSK9, promote LDLR recycling and lowers LDL-C levels. In addition, an LDL-R mimetic with the gain-of-function H306Y substitution which increases the affinity of the receptor for PCSK9 is in preclinical development as other peptide candidates (109). Pep2-8 is also the small peptide that mimics the secondary structural elements of the EGF-A domain, hindering the interaction between PCSK9 and the LDLR (110). Alternatively, approaches involving the catalytic domain, prodomain, or C-terminal domain of PCSK9 can be employed. It has been demonstrated that mimetics derived from PCSK9 fragments reduce PCSK9-mediated degradation of LDLR *in vivo* (111, 112).

Annexin A2 (AnxA2) is an extracellular endogenous antagonist that interacts with the C-terminal domain of PCSK9, and has received attention as a natural inhibitor of PCSK9 binding to LDLR (113, 114). High levels of AnxA2 are present in the lung, pancreas, colon, and adrenal tissues, while low levels of this antagonist are present in the liver, kidney, and spleen. The repeat-one domain of AnxA2 can specifically bind to the C-terminal domain of PCSK9 at the cell surface and inhibit PCSK9-mediated degradation of the LDLR. In AnxA2 knock-out mice, there was an ~2-fold increase in the circulating PCSK9 levels and an ~1.4-fold increase in LDL-C levels was observed, in addition to a reduction of LDLR protein levels by ~50% in extrahepatic tissues. On the other hand, an adenoviral overexpression of AnxA2 in the liver results in an increase of LDLR protein levels, mostly in extrahepatic tissues (114). Furthermore, a peptide mimicking AnxA2 repeat-one domain directly inhibited the interaction between PCSK9 and LDLR (115). Thus, the extrahepatic physiological role of AnxA2 in the regulation of PCSK9 enhances the degradation of LDLR. A small peptide mimicking AnxA2 may constitute a potential approach for PCSK9 inhibition.

#### Adnectin

Adnectin is a synthetic protein based on the 10th type III domain of human fibronectin. Its variable loops can be designed to efficiently introduce a surface that binds therapeutically relevant targets with high affinity and specificity (116). It is small and compact, with no sequence homology to immunoglobulins, although it possesses a β-sheet–fold structure with diversified loops analogous to the antibody variable regions (117, 118). Adnectin BMS-962476 (by Bristol-Myers Squibb/Adnexus) is a PCSK9-targeting polypeptide conjugated with polyethylene glycol to enhance its pharmacokinetic profile, which binds human PCSK9 with a subnanomolar affinity. Adnectin hinders the interaction between extracellular PCSK9 and the EGF-A domain of LDLR as a potential alternative to MAbs, and prevents PCSK9-induced degradation of the LDLR. Its production is easier and less expensive than that of antibodies since the molecule can be produced using bacterial expression systems. A preclinical trial revealed that BMS-962476 (5 mg/kg) rapidly reduces free PCSK9 levels by more than 99%, while increasing 6-fold the total PCSK9 levels in the cynomolgus monkey. Hence, LDL-C levels were reduced by up to 55% within 48 h, with the effects persisting for up to 3 weeks (119). In the first study involving humans, a single ascending regimen subcutaneous or intravenous dose of BMS-962476 resulted in a reduction of free PCSK9 levels by more than 90% without serious adverse events; LDL-C levels were reduced by up to 48% at maximum dose between day 4 and 14 (120). These observations suggested that BMS-962476 is a well-tolerated and effective agent without notable safety issues at an early phase of development. However, its effect should be followed over a longer time period in a large number of subjects. Nevertheless, BMS-962476 is a promising therapeutic drug and an alternative to MAbs against circulating PCSK9 through which to achieve significant lowering of LDL-C levels.

#### PCSK9 Vaccination

As an alternative strategy of long-term PCSK9 inhibition, a peptide-based anti-PCSK9 vaccination approach has been developed. PCSK9 vaccine stimulates the immune system to generate high-affinity PCSK9-specific antibodies, and then blocks the ability of PCSK9 to bind to the LDLR. Even though active vaccination exerts the same therapeutic effect as passive administration of PCSK9 MAbs, the effect can be achieved with fewer injections, at a lower dose, and potentially in a cost-effective manner, without the possibility of inducing a drug-neutralizing immune response. Induction of an antibody response against PCSK9 is limited by B-cell tolerance; however, efficient activation of self-reactive B-cells is provoked by the presence of foreign T-helper epitopes or antigen multivalency (121), which induce particularly robust, high-titer, and long-lasting autoantibody response against foreign antigens. A peptide-based anti-PCSK9 vaccine called AT04A consists of short peptides (by Affiris), mimicking fragments of a mature human PCSK9 protein, conjugated to a foreign carrier protein (Keyhole limpet hemocyanin) that provides T-helper cell epitopes (122). The amino acid sequence of mimotope-peptides used for vaccination is different from the native peptide sequence to be targeted, but these peptides are still able to induce a highly PCSK9-specific antibody response. These peptides are recognized as foreign by the immune system because they do not share sequence identity with other human proteins, and offer a potent means of inducing specific antibodies to self-antigens. For instance, the total cholesterol and LDL-C levels were reduced by up to 30 and 50% in anti-PCSK9 immunized mice, respectively. The atherosclerosis was reduced by 64% in blood vessels with this approach. Moreover, the vaccine-generated anti-PCSK9 antibody had a half-life of ~4 months and resulted in sustained significant reductions in cholesterol levels for at least 1 year in mice. Booster vaccination with the same dose a year after the initial immunization effectively reactivated the anti-PCSK9 antibody response, revealing the potential of a yearly re-boosting immunization (123).

One approach for inducing a strong antibody response against self-antigens involves the displaying of self-antigen in a highly dense, repetitive format on the surface of virus-like particles (VLP). VLPs are formed by a self-assembly of the viral structure protein without a viral nucleic acid, which is used to produce multivalent vaccines. PCSK9-dysplaying VLPs elicit high-titer peptide-specific PCSK9-reactive response in mouse. A VLP-based vaccine targeting PCSK9 synergistically has been shown to reduce LDL-C levels by 30–40% in macaques treated with statins (124).

These findings suggest that PCSK9 vaccination could be a promising strategy for sustained reduction of LDL-C levels. However, longer-term safety, in addition to efficacy, is an important concern for its successful development. It is possible that active immunization would cause antibody-dependent cell-mediated cytotoxicity or complement-dependent cytotoxicity with non-specific cell destruction (125). A previous study demonstrated that PCSK9 vaccine is well-tolerated in short- and long-term experiments, and that immunization does not induce any apparent side effects (123). Further clinical studies are necessary to identify the optimal immunization scheme and to evaluate long-term safety. Accordingly, phase I clinical trials (AT04A and AT06A) are currently ongoing to test that.

### Inhibition of PCSK9 Secretion

PCSK9 is synthesized and transported from the ER to the Golgi apparatus, and is subsequently secreted into circulation. A loss-of -function mutation in the prodomain, results in an inhibition of the secretion of PCSK9 because protein trafficking is disrupted (25). Inhibition of pathways which are major mediators of PCSK9 secretion may prevent PCSK9 from reaching the cell surface, which may in turn be used for preserving LDLR expression in the liver and subsequent reduction of plasma LDL-C levels.

#### Small Molecules

Sortilin is encoded by the gene SORT1, which is a high-affinity sorting receptor of PCSK9 and thus influences cholesterol levels. It predominately co-localizes with PCSK9 in the trans-Golgi network. Sortilin facilitates cellular secretion of PCSK9 in the late secretory pathway from the trans-Golgi network to the plasma membrane (126). The strongest interaction between sortilin and PCSK9 occurs at a pH 6.5, whereas it is completely lost at pH 5.5. Thereby, sortilin binds PCSK9 in the trans-Golgi network (pH ~6.5) and releases it in secretary vesicles (pH ~5.5). Increased intracellular PCSK9 levels and reduced circulating PCSK9 levels occur in sortilin-deficient mice. These effects increase LDLR levels in the liver and reduce plasma LDL-C levels. On the other hand, overexpression of human sortilin in the liver increases circulating PCSK9 levels and reduces hepatic LDLR levels, thus increasing plasma LDL-C levels. Moreover, a significant positive correlation between sortilin and PCSK9 is observed in human cohort studies. In healthy individuals, a significant positive correlation between sortilin and PCSK9 was noted, suggesting that sortilin expression affects circulating PCSK9 levels (126, 127). In addition, sortilin is associated with not only dysregulated lipoprotein metabolism but also the development of type 2 diabetes mellitus and progression of atherosclerosis. These findings suggest that sortilin might be a potential therapeutic target to reduce atherogenic risk. However, sortilin is essential for proper neuronal functionality on the nervous system. Therefore, caution is needed when considering the use of sortilin as a therapeutic tool (128, 129).

Sec24a, a protein which is incorporated into the coat protein complex II-coated vesicles, is required for the transport of PCSK9 from the ER to the Golgi apparatus. In Sec24a-deficient mice, reduced plasma PCSK9 levels and elevated hepatic PCSK9 levels are observed, which lead to an increase in LDLR expression on the liver surface. In addition, overexpression of Sec24a promotes the secretion of PCSK9 (130, 131). The pharmacological inhibition of Sec24a function may be a useful alternative potential approach to inhibit PCSK9 secretion. However, it is worth noting that it is important to identify the presence of other proteins that require Sec24a for their secretion from the ER.

SRT3025 is a third-generation synthetic Sirtuin-1 (SIRT1)-activating compound, currently under investigation as an allosteric activator of SIRT1 (by GSK/Sirtis). SIRT1, a member of the sirtuin family of NAD ± dependent deacetylases, contributes to the regulation of body energy homeostasis. It is broadly expressed in many tissues including adipose tissue, liver, pancreas and skeletal muscle. Therefore, SIRT1 plays also an essential role in the regulation of hepatic lipid metabolism (132). *In vitro* SRT3025 attenuates PCSK9 secretion from hepatocytes and reduces plasma PCSK9 levels *in vivo*, and thus increases LDLR expression in the hepatocytes. This is accompanied by a significant reduction of plasma LDL-C levels and atherosclerosis (133). Administration of SIRT1 to elderly individuals was safe, and serum cholesterol, LDL-C, and triglyceride decreased (134). Employing a pharmacological SIRT1 activator such as SRT3025 might offer a potential anti-atherosclerotic strategy.

Quercetin is a phytochemical which occurs predominantly as quercetin-3-O-beta-glucoside (Q3G) in a broad range of fruits and vegetables. Oral administration of Q3G was shown to have cholesterol lowering and insulin lowering properties (135, 136). Q3G has been found to reduce PCSK9 secretion and to increase LDLR expression, leading to increased LDL-C uptake and reduction in plasma LDL-C levels. Moreover, it was reported that Q3G does not affect SEC24A expression, but significantly inhibits intracellular sortilin level (by 50%) and its m-RNA level (by 40%) (137). However, another report suggested that Q3G does not affect PCSK9 expression in mice fed a low-cholesterol diet; in mice fed a high-cholesterol diet, reduction of circulating PCSK9, and a corresponding increase in cellular PCSK9 and LDLR levels was observed (136). This might be caused by a corrective activity of Q3G, in that Q3G reverses the LDLR/PCSK9 ratio in the liver. These observations suggest that Q3G supplementation on the background of a high-cholesterol diet results in regulation of PCSK9 expression and secretion, by inhibiting sortilin expression. This results in LDL-C clearance by increasing hepatic LDLR, and reversing hypercholesterolemia caused by the diet. Further studies on its effects on lipid and glucose metabolism may offer a chance of developing a therapy effective in high-risk patients with dyslipidemia and diabetes mellitus.

PCSK9 sequence variation resulting from the PCSK9-Gln^152^/His substitution was identified in a French-Canadian family, which results in restricted proteolysis and secretion through separate mechanisms through separate mechanisms. It was reported that the loss-of-function PCSK9-Gln^152^/His variant is associated with reduced circulating PCSK9 and LDL-C levels (by 79 and 48%, respectively). In addition, the Gln^152^/His amino acid substitution reduces the ability of proPCSK9 to undergo autocatalytic cleavage and subsequent secretion *in vitro* (138, 139). These findings support the notion that inhibition of autocatalytic processing in the ER may be a novel strategy to specifically alter PCSK9 secretion.

## Future Perspectives and Controversies

Statins are the first-line drug treatment for hypercholesterolemia; however, even the maximally tolerated dose of statin, alone or in combination with ezetimibe, is not sufficient for attaining the LDL-C goals in patients with the highest risk and most extreme levels of cholesterol. Therefore, PCSK9 inhibitors have attracted a lot of attention as a potent option for regulating LDL-C levels. PCSK9 expression is regulated by a SREBP-2 transcription factor, which induces the expression of genes involved in cholesterol synthesis and uptake, according to the intracellular cholesterol levels (34). Statins induce SREBP-2 activity by inhibiting the 3-hydroxy-3methylglutaryl coenzyme A reductase. This leads to both a reduction of endogenous cholesterol synthesis and an increase in LDLR expression (Figure 1). However, statins also enhance PCSK9 expression by activating SREBP-2, which attenuates the reduction of plasma LDL-C levels by degrading LDLR. This mechanism at least in part explains the observation that doubling of a statin dose only results in an additional 6% reduction of LDL-C levels. This suggests that adding PCSK9 inhibitors to a statin therapy may result in a beneficial synergistic effect (140). It was reported that as many as 20% of patients who received statin therapy exhibited statin-associated muscle symptoms in a routine care setting. Statin intolerance greatly contributes to the burden of CVD together with patient related factors such as medication discontinuation and low adherence (141, 142). Therefore, inhibiting PCSK9 may be a useful alternative approach in high-risk patients with statin intolerance.

Each approach to PCSK9 inhibition has distinct advantages and disadvantages (Table 1). Even though the use of anti-PCSK9 MAbs can lead to a remarkable reduction in LDL-C levels and an acceptable risk of adverse events, their inconvenience on account of 12 or 24 yearly subcutaneous injections and high manufacturing costs are major impediments to practical use. On the other hand, the new approaches to PCSK9 inhibition may offer the possibility to improve on the durability of effect, route of administration, adherence, storage, and cost effectiveness, even though none of these have been approved. For example, small molecules can be administered orally without adverse events at the injection site, including pain. Thus, they might be broadly more acceptable to patients. In addition, the productions of these inhibitors are easier and cheaper than those of anti-PCSK9 MAbs. Thus, oral administration in combination with statins may be an attractive strategy. One advantage of siRNA and vaccination over anti-PCSK9 MAbs is the infrequency of administrations (once or twice a year vs. 12–24 injection per year), which not only offers the potential of significant and consistent reduction of LDL-C levels, but also improvement in adherence and, subsequently, low inter-patient variability in response to treatment due to non-compliance. The manufacturing cost of inclisiran is assumed to be lower than that of anti-PCSK9 MAbs and the same as that of small molecules because of the relatively easy manufacturing process and stability of dried oligonucleotides at room temperature (66). PCSK9 vaccination is also likely to be cheaper than anti-PCSK9 MAbs and easy to apply to large populations in most countries (125). These promising approaches to PCSK9 inhibition are expected to provide potential benefits beyond those of anti-PCSK9 MAbs.

Anti-PCSK9 MAbs inhibit only the extracellular PCSK9 activity but, on the other hand, some approaches focused on how PCSK9 expression and secretion affect intracellular PCSK9 activity. For example, inclisiran reduces the intracellular and extracellular PCSK9 levels by inhibiting PCSK9 synthesis in the hepatocytes. However, the magnitude of the effect of inclisiran on all lipid and lipoprotein levels is broadly similar to that of anti-PCSK9 MAbs (67, 68). This suggests that the inhibition of intracellular PCSK9 may not contribute meaningfully to the regulation of PCSK9-mediated LDLR degradation, which is dependent on the PCSK9 in the circulation. Nevertheless, inclisiran has a long-lasting and durable effect of LDL-C reduction compared to anti-PCSK9 MAbs after a single injection and this may be associated more with the effect of altering intracellular PCSK9 metabolism or quite simply mean that the inclisiran RISC complex is highly stable and catalytic and hence can degrade multiple transcripts. Since the function and mechanism of intracellular PCSK9 inhibition remain unclear, additional lipoprotein kinetic studies would help to reveal the effect of the inhibition of intracellular pathway on lipid metabolism. In addition, relevant kinetic studies of other approaches including the inhibition of secretion will be helpful to understand the potential role of PCSK9 in the intracellular pathway.

New therapeutic strategies with novel mechanisms of action are being developed, which appear to be reasonably effective. However, the successful development entirely depends on their safety (143, 144) (Table 2). The efficacy of PCSK9 MAbs and loss-of-function mutation of PCSK9 revealed that very low LDL-C levels had no major safety concern (28, 145, 146). Nevertheless, PCSK9 has other possible physiological roles in the inflammatory response, glucose metabolism, and the nervous system beyond lipid metabolism. Several studies reported that it causes insulin intolerance, progression of lesion inflammation, and neurogenesis or neuronal apoptosis (147). EBBINGHAUS (Evaluating PCSK9 Binding antiBody Influence oN cognitive in HeAlth cardiovascular Risk Subjects) evaluated the possible adverse effects of evolocumab on cognition in 1974 patients from the FOURIER trial, but found no evidence of statistically significant adverse effects on cognition (148). In contrast, meta-analysis of data from more than 10,000 patients receiving PCSK9 MAbs raised the possibility through adverse event reporting of an increased risk of cognitive impairment, which was unrelated to achieved LDL-C levels (149–151). The new therapeutics to inhibit PCSK9 may be associated with unexpected off-target effects within the same or different organs and the long-term safety of new mechanisms of action and technologies is still unknown. Therefore, further studies are required to elucidate the global physiological function of PCSK9; improve our understanding of the mechanisms by which each approach reduces PCSK9; and long-term assessment of safety. Should the safety be proven, these will pave the way for additional potent therapeutic options for the reduction of LDL-C.

**Table 2:** The adverse effects in clinical trials.

	**Compounds**	**Any AEs**	**SAEs**	**Injection site reaction**	**Laboratory abnormality**	**References**
MAbs*	Alirocumab	1942/2476 (78.4%) **	387/2476 (15.6%)	183/2476 (7.4%)	AST, ALT > 3× ULN: 1.4%, 1.8%	(143)
		vs. 1004/1276 (78.7%)	vs. 205/1276 (16.1%)	vs. 67/1276 (5.3%)	CK > 3× ULN: 3.6%
	Evolocumab	2016/3946 (51.1%)	110/3946 (2.8%)	131/3946 (3.3%)	AST or ALT > 3× ULN: 0.4%	(144)
		vs. 1031/2080 (49.6%)	vs. 43/2080 (2.1%)	vs. 63/2080 (3.0%)	CK > 5× ULN: 0.7%
siRNA*	Inclisiran	282/370 (76.2%)	41/370 (11.1%)	19/370 (5.1%)	AST, ALT > 3× ULN: 0.3%, 0.8%	(67)
		vs. 96/127 (75.6%)	vs. 9/127 (7.1%)	vs. 0/127 (0%)	CK > 5× ULN: 1.4%
ASO	SPC5001		Acute kidney injury	8/18 (44%)	Serum creatinine 18% increase	(75)
Adnectin	BMS-962476	31/64 (48.4%)	2/64 (3.1%)		No remarkable findings	(120)
			Cerebrovascular event		

* *vs. Placebo*.

** *Treatment emergent adverse events*.

*AEs, adverse events; SAEs, serious adverse events; ULN, upper limit of normal. MAbs, monoclonal antibodies; ASO, antisense oligonucleotide; siRNA, small interfering RNA. AST, aspartate aminotransferase; ALT, alanine transaminase; CK, creatine kinase*.

## Author Contributions

TN searched the literature and wrote the manuscript. KR provided guidance and edited the manuscript.

### Conflict of Interest Statement

KR has received personal fees (data safety monitoring board) from AbbVie, Inc.; consultant fees/honoraria from Aegerion, Algorithm, Amgen, AstraZeneca, Boehringer Ingelheim, Cerenis, Eli Lilly and Company, Ionis Pharmaceuticals, Kowa, Medicines Company, MSD, Novartis, Pfizer, Regeneron Pharmaceuticals, Inc., Resverlogix, Sanofi, and Takeda; and research grants from Kowa, Pfizer, and Regeneron Pharmaceuticals, Inc. CD, MB-Bobanovic. TN was supported by a grant from the Uehara Memorial Foundation.

## Abbreviations

AnxA2: annexin A2
ASO: antisense oligonucleotide
BBR: berberine
CETP: cholesteryl ester transfer protein
Cas9: CRIPSR-associated protein 9
CRISPR: clustered regularly interspaced short palindromic repeat
CVD: cardiovascular disease
EGF: epidermal growth factor
ER: endoplasmic reticulum
HNF: hepatocyte nuclear factor
LDL: low-density lipoprotein
LDL-C: LDL cholesterol
LDLR: LDL receptor
MAb: monoclonal antibody
PCSK9: proprotein convertase subtilisin kexin 9
Q3G: quercetin-3-O-beta-glucoside
sgRNA: single guide RNA
siRNA: small-interfering RNA
Sirt1: Sirtuin-1
SRE: sterol regulatory element
SREBP: sterol regulatory element-binding protein
VLP: virus-like particle.
